# Ethnic comparison of pharmacokinetics of ^18^F-florbetaben, a PET tracer for beta-amyloid imaging, in healthy Caucasian and Japanese subjects

**DOI:** 10.1007/s00259-014-2890-8

**Published:** 2014-08-21

**Authors:** Michio Senda, Masahiro Sasaki, Tomohiko Yamane, Keiji Shimizu, Marianne Patt, Henryk Barthel, Bernhard Sattler, Toshiki Nagasawa, Marcus Schultze-Mosgau, Yasuko Aitoku, Ludger Dinkelborg, Osama Sabri

**Affiliations:** 1 Division of Molecular Imaging, Institute of Biomedical Research and Innovation, 2-2 Minatojima-Minamimachi, Chuo-ku Kobe, 650-0047 Japan; 2 Department of Nuclear Medicine, University of Leipzig, Leipzig, Germany; 3 Bayer Yakuhin Ltd, Osaka, Japan; 4 Bayer HealthCare AG, Berlin, Germany; 5 Piramal Imaging GmbH, Berlin, Germany

**Keywords:** Florbetaben, PET, Alzheimer’s disease, Beta-amyloid, Pharmacokinetics

## Abstract

**Purpose:**

^18^F-Florbetaben is a positron emission tomography (PET) tracer indicated for imaging cerebral beta-amyloid deposition in adult patients with cognitive impairment who are being evaluated for Alzheimer’s disease and other causes of cognitive decline. The present study examined ethnic comparability of the plasma pharmacokinetics, which is the input to the brain, between Caucasian and Japanese subjects.

**Methods:**

Two identical phase I trials were performed in 18 German and 18 Japanese healthy volunteers to evaluate the plasma pharmacokinetics of a single dose of 300 MBq ^18^F-florbetaben, either of low (≤5 μg, LD) or high (50–55 μg, HD) mass dose. Pharmacokinetic parameters were evaluated based on the total ^18^F radioactivity measurements in plasma followed by metabolite analysis using radio-HPLC.

**Results:**

The pharmacokinetics of ^18^F-florbetaben was characterized by a rapid elimination from plasma. The dose-normalized areas under the curve of ^18^F-florbetaben in plasma as an indicator of the input to the brain were comparable between Germans (LD: 0.38 min/l, HD: 0.55 min/l) and Japanese (LD: 0.35 min/l, HD: 0.45 min/l) suggesting ethnic similarity, and the mass dose effect was minimal. A polar metabolite fraction was the main radiolabelled degradation product in plasma and was also similar between the doses and the ethnic groups.

**Conclusion:**

Absence of a difference in the pharmacokinetics of ^18^F-florbetaben in Germans and Japanese has warranted further global development of the PET imaging agent.

## Introduction

Positron emission tomography (PET) imaging of cerebral beta-amyloid deposition is valuable for adult patients with cognitive impairment who are being evaluated for Alzheimer’s disease (AD) and other causes of cognitive decline as well as for evaluation of treatments aimed at reducing amyloid burden. While ^11^C-Pittsburgh compound B (PIB) has been most widely used as a PET imaging agent of cerebral beta-amyloid deposition, the short half-life of ^11^C (*T*_1/2_ = 20 min) reduces clinical practicality and has prompted development of a number of PET imaging agents labelled with ^18^F (*T*_1/2_ = 110 min), including ^18^F-florbetaben or trans-4-(*N*-methyl-amino)-4′-{2-[2-(2-[^18^F]fluoro-ethoxy)-ethoxy]-ethoxy}-stilbene (previously also known as BAY 94-9172). Initial clinical studies have revealed promising results regarding the diagnostic capability of ^18^F-florbetaben as it accumulated in the cerebral cortex of patients with AD, while very low uptake was observed in normal subjects and in patients with frontotemporal dementia [1, 2]. Those reports triggered global clinical development of ^18^F-florbetaben as a PET diagnostic agent [3], and the initial results were confirmed in a pivotal histopathology study [4], leading to recent approval in Europe and the USA (NeuraCeq™).

Plasma pharmacokinetics, which is the input function to organs, is of importance for evaluation of the brain uptake of the PET agent, especially in cases of kinetic analysis of the brain time-activity curves [5]. Because ethnicity may affect the pharmacokinetics of a drug [6, 7], it is essential to confirm similar plasma pharmacokinetics of ^18^F-florbetaben in different ethnic populations as a basis for further global development on multiple races and ethnicities that are designed to evaluate the brain accumulation in various patients and normal subjects. The present report compares the plasma time course of ^18^F-florbetaben and its labelled metabolites, blood to plasma ratio and urinary excretion following intravenous injection in healthy German and Japanese subjects.

## Materials and methods

### Study design

Two identical studies were carried out as phase I clinical trials of ^18^F-florbetaben in healthy subjects which were conducted at the University of Leipzig, Leipzig, Germany and at the Institute of Biomedical Research and Innovation (IBRI), Kobe, Japan. The pharmacokinetic data of plasma and urine were acquired as well as the safety data. Also the radioactivity accumulation and distribution in the brain and the whole body was imaged using a PET camera, the results of which will be reported in separate publications.

At each site, a total of 24 subjects were recruited. The first 12 subjects were intravenously injected with either 300 ± 60 MBq of ^18^F-florbetaben of low mass dose (LD: <5 μg) (*n* = 9) or placebo (PL: vehicle) (*n* = 3), in a single-blind random manner. After safety data were assessed and the sponsor and the investigator confirmed that no safety concerns had been raised, the latter 12 subjects were intravenously injected with either 300 ± 60 MBq of ^18^F-florbetaben of high mass dose (HD: >50 and ≤55 μg) (*n* = 9) or placebo (vehicle) (*n* = 3), in a single-blind random manner. The HD study was designed to examine the mass dose effect on pharmacokinetics and to demonstrate the practicability of delivery of ^18^F-florbetaben from a production site to distant PET imaging sites, for which product vials containing higher radioactivity are produced to compensate for the radioactive decay during the transportation. As a result a higher amount of coexisting non-radioactive florbetaben remains as a high mass dose.

All studies were conducted based on Good Clinical Practice in accordance with the Declaration of Helsinki and its revisions. At each site, the study protocol was reviewed and approved by each Institutional Review Board (University of Leipzig Ethics Committee and IBRI Institutional Review Board). All subjects provided written informed consent before participating in the study after the study procedures had been fully explained both orally and in written form.

### Subjects

The participants were either of German or Japanese ethnicity and were healthy subjects aged greater than or equal to 55 years, and women were without child-bearing potential. They underwent screening tests that covered medical and family history, physical and neurological examination, ECG, blood and urine test, neuropsychological tests and a brain MRI scan. They were eligible if the neuropsychological scores were within the normal range and if the MRI was age appropriate except for very mild medial temporal lobe atrophy and mild to moderate age-related white matter lesions.

Exclusion criteria were a history of significant psychiatric or neurological illness, history of drug or alcohol abuse, cancer within 5 years, positive test for human immunodeficiency virus (HIV), hepatitis B or hepatitis C virus or syphilis and any significant or unstable medical conditions such as unstable angina, recent myocardial infarction, cardiac failure, chronic renal failure, chronic liver disease, severe pulmonary disease, blood disorders, poorly controlled diabetes and chronic infection. They were also ineligible if the haematological or biochemical parameters were outside the normal range and considered clinically significant by the investigator.

### Synthesis of ^18^F-florbetaben

^18^F-florbetaben was manufactured and quality tested based on Good Manufacturing Practice for investigational drugs at each study site, which was audited by Bayer HealthCare. Briefly, ^18^F was produced by bombardment of ^18^O-enriched water with protons using an in-house cyclotron and trapped in a cartridge and was then eluted with Kryptofix K2.2.2 and K_2_CO_3_ into a reaction vessel. The precursor compound (BOC-stilbene mesylate) was added and the mixture was heated for ^18^F labelling, followed by cooling and hydrolysis [8]. On purification of ^18^F-florbetaben with radio-HPLC of the reaction mixture, the product fraction was fixed on a solid phase extraction cartridge, which was subsequently eluted with ethanol into the formulation. For the HD study, 50 μg of standard non-radioactive florbetaben was added to the formulation. For the LD study, no carrier was added. For the PL study, the injection was formulated in the same way without ^18^F-florbetaben.

The study drug was tested for quality. Identification of ^18^F was tested with gamma spectroscopy and half-life measurement, and the radionuclidic purity was ≥99 %. Identification of ^18^F-florbetaben was tested using HPLC with florbetaben as the reference standard, and the radiochemical purity was specified as ≥90 %, the actual value being 94.1 ± 1.6 % for Germans and 92.9 ± 1.6 % for Japanese. The pH was between 4.5 and 8.5. Residual solvents were specified as methanol ≤3,000 μg/ml, acetonitril ≤410 μg/ml and dimethyl sulphoxide (DMSO) ≤5,000 μg/ml. Residual Kryptofix 222 was <50 μg/ml.

### Pharmacokinetic assessments

The study drug (LD, HD or PL) was administered to the subjects as a slow intravenous injection to minimize the effect of ethanol, lasting 1 min in Germans and 2 min in Japanese, followed by a 10-ml saline flush. Venous blood was sampled before the injection and 2.5, 5, 10, 20, 30, 50, 70, 90, 120, 180, 240 and 360 min after start of injection for measurement of radioactivity concentration of whole blood and plasma using a gamma counter as well as for the analysis of labelled metabolites in plasma using radio-HPLC. Venous blood was also sampled in addition at 480, 720 and 1,440 min post-injection (p.i.) for radioactivity measurement only. In the Japanese study, metabolite analysis was omitted for the 5-, 20-, 50- and 90-min samples. The sampled blood volume was 2 ml for radioactivity measurement with additional 5 ml for metabolite analysis.

The blood samples in the German study were processed as described previously [9]. Briefly, 5 ml blood was centrifuged at 2,500 *g* for 5 min to generate plasma and then submitted to protein precipitation by addition of 2 volumes CH_3_CN and centrifugation at 6,000 *g* for 10 min. The resulting supernatant was analysed by HPLC [column Luna Phenyl-Hexyl 250 × 10 mm, 5 μm, flow 6 ml/min, injection volume >5 ml, gradient 20 % CH_3_CN (80 % water) increasing to 90 % at 12.1 min, increasing to 100 % at 13 min, 20 % at 13.1 min and 20 % at 15 min]. Essentially the same technique was used in the Japanese study except that each of the two centrifugation processes was done at 4,200 *g* for 10 min. The HPLC radiodetector in the German study was 3 × 3” NaI detector in combination with GABI star from Raytest (Straubenhardt, Germany), while that in the Japanese study was US2000 (1 in. diameter NaI) of Universal Giken (Kanagawa, Japan).

Urine was collected in the intervals 0–135, 135–300, 300–435 (for Japanese only) and 435–700 min for measurement of volume and radioactivity concentration using a gamma counter. Aliquots of the first two time intervals (135 and 300 min p.i.) were also analysed for radioactive metabolites using radio-HPLC in the same way as described above to obtain the fraction of ^18^F-florbetaben and labelled metabolites.

The plasma radioactivity data were corrected for the radioactive decay to obtain both the total ^18^F radioactivity concentration and the ^18^F-florbetaben radioactivity concentration after correction for the labelled metabolites at each time point. Then descriptive pharmacokinetic parameters were derived, including maximum plasma concentration normalized by the injected activity (C_max_/D), area under concentration vs time curve from zero up to the last measurable data point normalized by the injected activity (AUC0-t_last_/D) and blood to plasma ratio of total radioactivity. The results were expressed as geometric mean and coefficient of variation (CV) and were compared between LD and HD as well as between Germans and Japanese. No mathematical kinetic modelling analysis was carried out.

Statistical tests were carried out using two-way analysis of variance (ANOVA) with an ethnicity by mass dose interaction term to examine the effects of ethnicity and mass dose on the logarithm of AUC0-t_last_/D of plasma total radioactivity and ^18^F-florbetaben concentration as well as on the logarithm of AUC of labelled polar metabolite per injected activity up to 120 min. The level of significance was set to *p* = 0.05 without consideration of multiple comparisons. No statistical tests were carried out on C_max_/D because it depends on the injection speed, which was not sufficiently controlled in this study.

The urinary radioactivity data were also analysed to compute the percentage urinary excretion of the injected ^18^F radioactivity both in the form of ^18^F-florbetaben and labelled metabolites for each interval of the urine collection.

### Safety assessments

Safety data were acquired on symptoms and signs, ECG and blood and urine test before and 6, 24 and 48 h p.i. and additionally on ECG at 2 h 15 min and 12 h.

## Results

### Participants

Table 1 summarizes the demographics of the subjects. All German subjects were Caucasian, and all Japanese subjects were Asian. While the age was comparable, the average body weight of the Japanese subjects was 19 % less than the Germans.

**Table 1:** Demographics and injections

Parameters	Germans	Japanese
Number of subjects (LD, HD, PL)	24 (9, 9, 6)	24 (9, 9, 6)
Sex, male/female	12/12	17/7
Age, mean (range)	62.4 (55–75)	61.5 (55–71)
Weight, kg, mean (range)	75.0 (52.0–92.2)	60.9 (42.0–75.7)
Height, cm, mean (range)	170 (155–185)	163 (145–177)
Mean injected activity (MBq) LD, HD, PL	309, 293, 0	309, 296, 0
Mean injected dose (μg) LD, HD, PL	0.8, 50, 0	2.5, 53.3, 0

*LD* low mass dose, *HD* high mass dose, *PL* placebo

### Pharmacokinetics

**Fig. 1:**
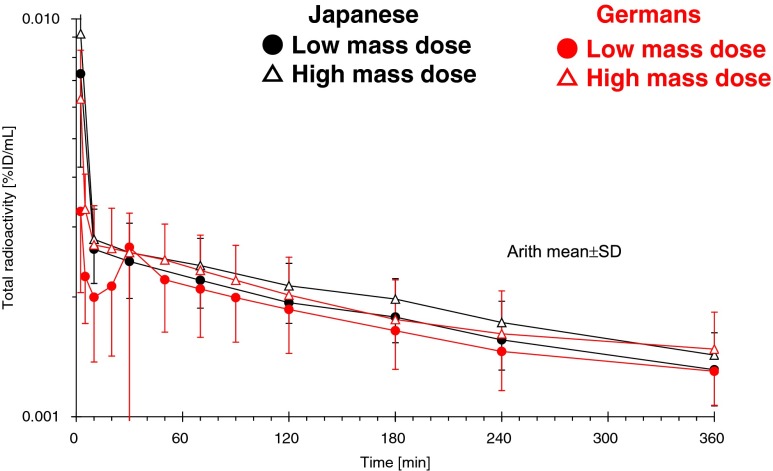
Total ^18^F radioactivity concentration time profile in plasma (decay-corrected)

The plasma concentration of total ^18^F radioactivity was at maximum at the first sampling time point (2.5 min) in most subjects and decreased rapidly thereafter. Figure 1 illustrates the total plasma ^18^F radioactivity curve for each ethnic group and for each mass dose. Table 2 presents the pharmacokinetic parameters derived from the total plasma ^18^F radioactivity concentration vs time curves. No substantial difference was observed between LD and HD. Japanese showed a higher C_max_/D value than the Germans possibly due to the longer injection duration in Japanese subjects. No significant difference was observed in AUC0-t_last_/D between the ethnicities or between LD and HD. No significant interaction effect between ethnicity and mass dose was observed.

**Table 2:** Pharmacokinetic parameters of total ^18^F radioactivity in plasma after single intravenous injection of ^18^F-florbetaben

	C_max_/D	AUC0-t_last_/D
	(%ID/l)	(%ID·h/l)
	Mean (CV)	Mean (CV)
Germans		
LD	3.48 (42.0 %)	10.1 (21.3 %)
HD	6.00 (34.0 %)	10.1 (37.8 %)
Japanese		
LD	6.74 (43.7 %)	10.9 (12.8 %)
HD	8.81 (45.8 %)	12.0 (12.6 %)

*ID* injected dose, *Mean* geometric mean, *CV* coefficient of variation, *LD* low mass dose, *HD* high mass dose

The ratio of the blood to plasma radioactivity concentration was 0.75 for Germans and 0.80 for Japanese at 2.5 min and gradually decreased toward 180 min, being approximately 0.7 at 70 min. No remarkable difference was observed between LD and HD or between the ethnicities.

**Fig. 2:**
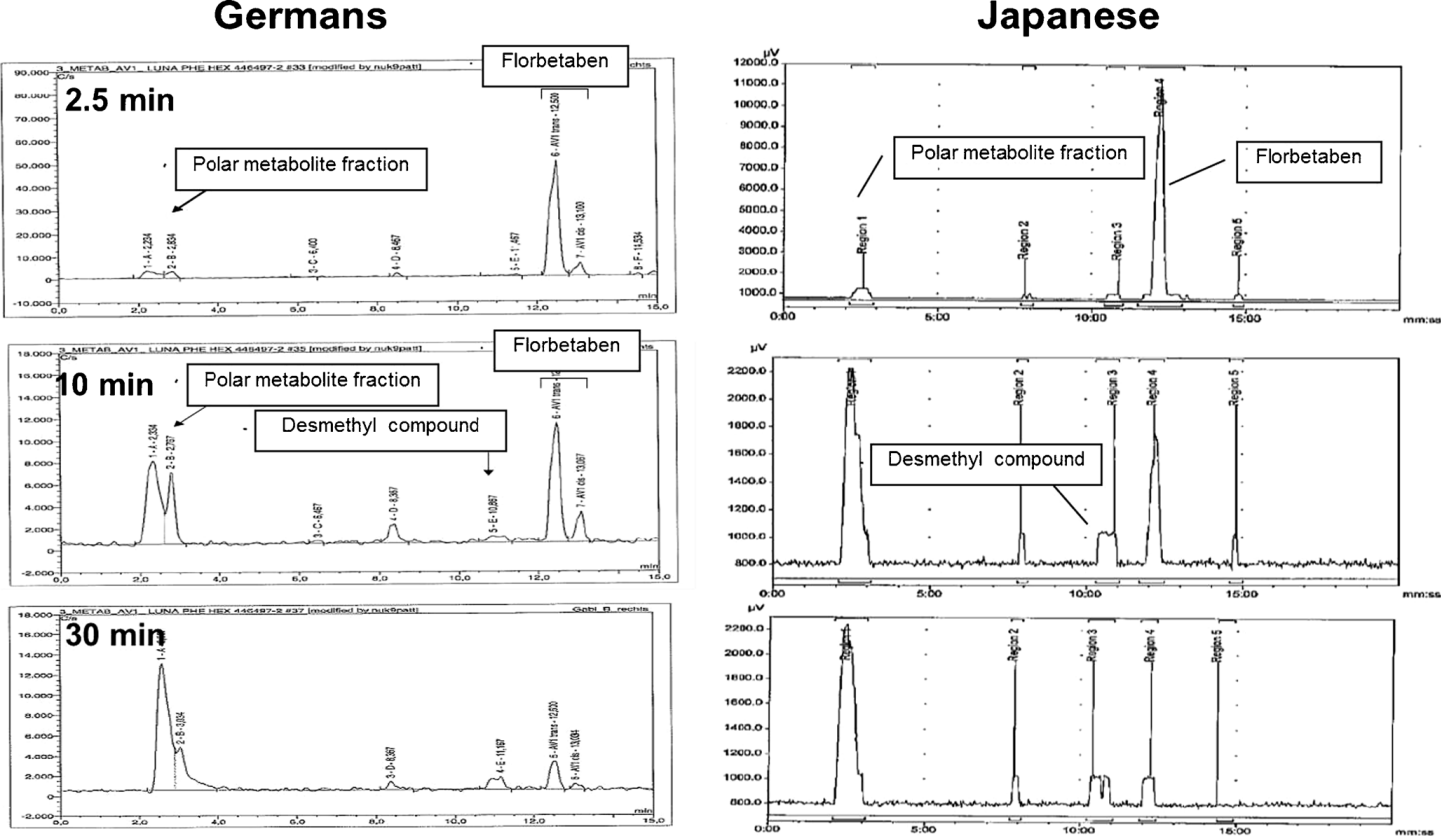
Representative HPLC radiochromatogram of the plasma samples at 2.5, 10 and 30 min p.i. of ^18^F-florbetaben in a German and Japanese subject

Figure 2 illustrates the radio-HPLC output of the plasma metabolite analysis. At 2.5 min p.i. most of the radioactivity in the plasma was ^18^F-florbetaben (on average, 83 and 78 % for LD and HD in Germans and 80 and 85 % for LD and HD in Japanese, respectively), which decreased rapidly at 30 min p.i. paralleled by an increase in the metabolite fraction (18 and 12 % for LD and HD in Germans and 8 and 11 % for LD and HD in Japanese). A polar metabolite fraction was the main radiolabelled degradation product in plasma. A small radioactivity peak was also detected that eluted directly prior to ^18^F-florbetaben both in Germans and Japanese and was identified as *N*-desmethyl ^18^F-florbetaben based on co-elution with an authentic reference standard in the German study.

**Fig. 3:**
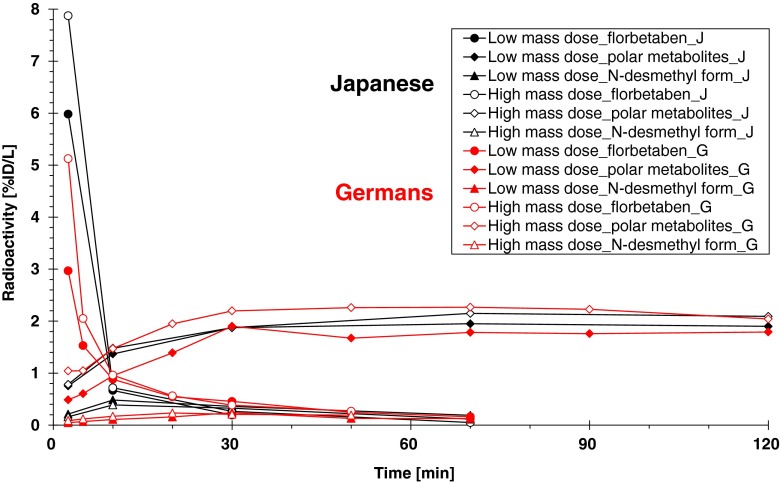
Mean plasma concentration time profiles of ^18^F-florbetaben and its metabolites (decay-corrected)

Table 3 represents the pharmacokinetic parameters of the plasma ^18^F-florbetaben concentration obtained from the data of the plasma parent fraction. Figure 3 depicts plasma concentration curves of unchanged ^18^F-florbetaben, ^18^F-labelled polar metabolites and *N*-desmethyl ^18^F-florbetaben for each ethnic group and for each mass dose. The pharmacokinetics of ^18^F-florbetaben was characterized by rapid elimination from plasma as ^18^F-labelled polar metabolites appeared and increased in the same time frame. When two-way ANOVA was carried out on the AUC0-t_last_/D of plasma ^18^F-florbetaben concentration (%ID/l), a small but significant difference (*p* < 0.05) was observed between LD and HD, while no significant difference was observed between Germans and Japanese. Statistical analysis on the AUC (0–120 min) of the ^18^F-labelled polar metabolite (Fig. 3) also showed a small but significant difference (*p* < 0.05) between LD and HD, while no significant difference was observed between Germans and Japanese. No significant interaction effect between ethnicity and mass dose was observed.

**Table 3:** Pharmacokinetic parameters of ^18^F-florbetaben in plasma after single intravenous injection of ^18^F-florbetaben

	C_max_/D	AUC0-t_last_/D
	(%ID/l)	(%ID·h/l)
	Mean (CV)	Mean (CV)
Germans		
LD	1.97 (41.3 %)	0.640 (32.0 %)
HD	4.64 (35.7 %)	0.924 (35.4 %)
Japanese		
LD	5.30 (59.3 %)	0.587 (44.3 %)
HD	6.46 (108 %)	0.745 (41.4 %)

*ID* injected dose, *Mean* geometric mean, *CV* coefficient of variation, *LD* low mass dose, *HD* high mass dose

Table 4 summarizes the urinary excretion of total ^18^F radioactivity, ^18^F-florbetaben and ^18^F polar metabolites expressed as per cent of injected activity for each interval of urine collection. Approximately 13–20 % of the injected radioactivity dose was excreted renally during the first 135 min. Almost all radioactivity in the urine was excreted as metabolites, whereas only trace amounts (0.1–0.2 %) of the radioactivity dose were detected as ^18^F-florbetaben in the first collection interval from 0 to 135 min. Of the detected metabolites, a polar metabolite fraction eluted from the column contributed to almost all radioactivity. This fraction was prominent in all collection intervals. Overall, approximately 26–36 % of the administered radioactivity dose was excreted in urine up to 12 h p.i. Only around 0.1 % of the dose was recovered as unchanged ^18^F-florbetaben in urine and excretion occurred only in the first collection interval (0–135 min) when ^18^F-florbetaben concentrations were highest in plasma. No ethnic difference or dose effect was observed in the urinary excretion.

**Table 4:** Urinary excretion of total ^18^F radioactivity, ^18^F-florbetaben and ^18^F polar metabolites expressed as per cent of injected activity (mean ± SD)

Interval	Total ^18^F		^18^F-florbetaben		^18^F polar metabolites	
	LD	HD	LD	HD	LD	HD
Germans						
0–135 min	12.7 ± 2.56 (9)	14.0 ± 4.14 (8)	0.194 ± 0.130 (7)	0.182 ± 0.063 (7)	11.6 ± 2.33 (7)	12.7 ± 4.18 (8)
135–240 min	4.94 ± 1.84 (9)	5.25 ± 1.85 (8)	0.033 ± 0.022 (3)	0.034 ± 0.026 (6)	4.38 ± 0.67 (7)	4.65 ± 1.63 (8)
0-t_last_^a^	26.4 ± 4.21 (9)	30.8 ± 6.77 (8)				
Japanese						
0–135 min	19.5 ± 7.9 (9)	17.4 ± 6.1 (9)	0.117 ± 0.038 (8)	0.111 ± 0.044 (9)	18.7 ± 7.6 (9)	16.6 ± 5.8 (9)
135–305 min	5.3 ± 2.9 (9)	8.2 ± 3.6 (9)	ND	ND	5.0 ± 2.7 (9)	7.9 ± 3.4 (9)
305–435 min	3.7 ± 1.1 (9)	4.9 ± 0.6 (9)				
435–720 min	6.2 ± 4.9 (8)	5.2 ± 1.3 (9)				
0–720 min	33.1 ± 7.2 (8)	35.7 ± 5.1 (9)				

Mean ± SD (*n*)

*ND* not detected, *LD* low mass dose, *HD* high mass dose

^a^Last collection time ranged between 5.0 and 12.3 h

### Safety

Most frequently reported treatment-emergent adverse events were related to injection site reactions including irritation, discomfort or pain observed in four PL, six LD and three HD German subjects and in four PL, eight LD and eight HD Japanese subjects (of six PL, nine LD and nine HD subjects, respectively, for both ethnicities). They were all mild and soon disappeared.

## Discussion

In the present study, German and Japanese healthy subjects were administered either LD or HD of ^18^F-florbetaben. The mean dose was 2.5 μg for LD and 53.3 μg for HD in the Japanese study and 0.8 μg and approx. 50 μg for the German study, respectively (Table 1). Thus, a factor of 20- to 60-fold difference in the mean mass dose was present between LD and HD.

After intravenous administration of 300 MBq of ^18^F-florbetaben to healthy subjects, maximum plasma concentrations of total radioactivity were observed at the first time point after the end of the infusion, i.e. at about 2.5 min after the start of the injection. Thereafter, total radioactivity concentrations declined rapidly in plasma, probably due to rapid and pronounced distribution into the whole-body tissues as well as due to metabolic clearance. Those findings are compatible with the previous report of the proof of mechanism study [9]. No difference was observed between LD and HD or between the ethnic groups (Fig. 1, Table 2).

A blood to plasma radioactivity ratio ranging 0.75 to 0.80 on averagewas observed at the first sampling time point of 2.5 min. This ratio was greater than 0.54 (corresponding to 1 minus measured haematocrit of 0.46), which might indicate that florbetaben weakly binds to or is distributed into red blood cells. There was a trend of a decreasing blood to plasma ratio up to 180 min p.i. This could be due to a different blood to plasma partitioning of the polar metabolite fraction, which represents almost all radioactivity in blood and plasma at later time points.

^18^F-Florbetaben was rapidly metabolized with appearance of labelled metabolites in plasma, the largest of which was one polar metabolite fraction that contributed to almost all radioactivity in plasma at later sampling time points (30 min p.i. and thereafter) (Fig. 2). This polar metabolite was observed to a similar extent in Germans and Japanese as well as for LD and HD injections. The structure of the polar metabolite is unknown but may result from cleavage of the ^18^F-labelled polyethylene glycol (PEG) side chain [9]. In addition to this polar metabolite peak, a minor radiopeak was detected on the radiochromatogram at a retention time between 10 and 11 min. Co-chromatography with an authentic reference standard suggests that this peak represents an *N*-desmethylated compound of ^18^F-florbetaben, which is the primary amine derivative of ^18^F-florbetaben. Overall, the concentrations of *N*-desmethyl ^18^F-florbetaben were small as compared to the polar metabolite fraction.

Plasma pharmacokinetics of ^18^F-florbetaben was obtained using information from the radio-HPLC metabolite fraction data and the total radioactivity data (Fig. 3), from which pharmacokinetic parameters were assessed (Table 3). Considering the methodological limitations of the quantification of a microdose amount of ^18^F-florbetaben (i.e. estimation based on the radio-HPLC and the total ^18^F radioactivity in plasma), the estimated concentrations and derived pharmacokinetic parameters provide only explorative and orienting information. Nevertheless, the evaluations are supportive to describe the basic pharmacokinetic properties of ^18^F-florbetaben since the plasma ^18^F-florbetaben concentration was followed up to the time point presenting 8 % or less of C_max_ in most subjects.

The dose-normalized C_max_ (C_max_/D) had a large variation with mean values showing an about twofold difference between the ethnicities or mass doses (Table 3), which is presumably related to the difference in the injection speed.

The AUC0-t_last_/D of plasma ^18^F-florbetaben concentration (%ID/l) showed a small (about 1.4-fold) but significant difference between LD and HD, while no difference was observed between Germans and Japanese. No significant difference was observed for AUC0-t_last_/D of plasma total activity concentration. Thus, there is a possibility of slight mass dose effect on the pharmacokinetics, although saturation of metabolic enzymes, such as cytochrome P450, is unlikely because it usually requires doses in the milligram or even gram range depending on the respective K_m_ values. In fact, the mean per cent of unchanged tracer relative to total ^18^F radioactivity in plasma decreased at a similar rate in both tracer mass dose groups at early sampling time points. Therefore, allowing for methodological limitations of the quantification, the present data indicate that the mass dose effect is minimal within a tracer mass dose range of up to 50 μg. Thus, the practicability of high mass dose injection is not impaired, which is relevant to remote delivery of the PET drug that requires a longer shipment time resulting in decreased specific radioactivity due to decay. In any case, the plasma concentration is cancelled out if the PET images are scaled to a reference region for visual interpretation or if the standard uptake value ratio (ratio of uptake to reference region) is used for quantitative evaluation of the cortical uptake.

In addition to plasma analysis, urine samples were analysed to estimate the proportion of radioactivity dose excreted in the urine. The data suggest that approx. 26–36 % of the injected activity was excreted renally up to 12 h. Almost the entire radioactivity was excreted as polar metabolites and only trace amounts of intact ^18^F-florbetaben were recovered in urine. The polar metabolite fraction, which was also the prominent species in plasma, represented the majority of radioactivity in urine, suggesting that these metabolites are excreted renally.

Lister-James et al. [10] have reported the pharmacokinetics of ^18^F-labelled florbetapir, which is another beta-amyloid tracer having a structure similar to ^18^F-florbetaben, with a benzene ring of ^18^F-florbetaben replaced by a pyridine ring. In their human study, ^18^F-labelled florbetapir rapidly cleared from blood leaving <5 % of injected activity in blood after 20 min, and the blood radioactivity derived mainly from polar metabolites by 90 min p.i. Urinary excretion of radioactivity was approximately 17 % of the dose by 200 min p.i. The pharmacokinetic results of ^18^F-florbetapir are similar to the data observed with ^18^F-florbetaben suggesting similar underlying pharmacokinetic processes for both tracers.

In the present study, venous plasma concentration of radioactivity and ^18^F-florbetaben was measured and the AUC was evaluated as a bulk input to the brain, which is an important factor for the radioactivity uptake by the brain in a later phase (e.g. 90 min p.i.) to be imaged for diagnosis of amyloid deposition [1]. Arterial plasma concentration was not measured because tracer kinetic analysis was out of the scope of the present study and accurate arterial input function was not necessary. In a previous study, arterial plasma concentration of radioactivity and ^18^F-florbetaben was measured and a representative case was reported [9]. The venous concentration obtained in the present study was comparable to the arterial data reported in the previous study except for the early phase (2.5 min p.i.) when the venous samples showed lower values than the arterial data due to dispersion. Furthermore, the ^18^F-florbetaben fraction of venous plasma radioactivity measured in the present study was very similar to those of arterial data [9].

### Conclusion

The pharmacokinetics of ^18^F-florbetaben is similar between German and Japanese subjects. After intravenous injection, ^18^F-florbetaben distributes into whole-body tissues and is rapidly eliminated from plasma. Within the methodological limits of quantification, the effect of mass dose on the pharmacokinetics of ^18^F-florbetaben is minimal up to about 50 μg in healthy subjects. A polar metabolite fraction constitutes the main radiolabelled degradation product in plasma. The present study confirmed ethnic similarity of ^18^F-florbetaben pharmacokinetics observed in plasma and urine as a basis of further global studies that evaluate and compare the brain accumulation in various races and ethnicities.
